# *Arabidopsis thaliana* DNA Damage Response Mutants Challenged with Genotoxic Agents—A Different Experimental Approach to Investigate the *TDP1α* and *TDP1β* Genes

**DOI:** 10.3390/genes16010103

**Published:** 2025-01-19

**Authors:** Anna Bertoncini, Paola Pagano, Anca Macovei

**Affiliations:** Department of Biology and Biotechnology “L. Spallanzani”, University of Pavia, Via Ferrata 9, 27100 Pavia, Italy; anna.bertoncini@unimi.it (A.B.); paola.pagano01@universitadipavia.it (P.P.)

**Keywords:** DNA damage response, camptothecin, tyrosyl-DNA phosphodiesterase1, NSC120686, gene expression profiles

## Abstract

**Background/Objectives:** DNA damage response (DDR) is a highly conserved and complex signal transduction network required for preserving genome integrity. DNA repair pathways downstream of DDR include the tyrosyl-DNA phosphodiesterase1 (TDP1) enzyme that hydrolyses the phosphodiester bond between the tyrosine residue of topoisomerase I (TopI) and 3′-phosphate end of DNA. A small TDP1 subfamily, composed of TDP1α and TDP1β, is present in plants. The aim of this work was to investigate the role of the two *TDP1* genes in the DDR context. **Methods:** A series of *Arabidopsis thaliana* DDR single and double mutants defective in the *sog1*, *e2fb*, *pol2A*, *atm*, and *atr* genes, treated with the genotoxic agents camptothecin (CPT, inhibitor of TopI) and NSC120686 (NSC, inhibitor of TDP1), were used. These compounds were specifically used due to their known impact on the TDP1 function. The effect of the treatments was assessed via phenotypic analyses that included germination percentage, speed, and seedling growth. Subsequently, the expression of the *TDP1α* and *TDP1β* genes was monitored through qRT-PCR. **Results:** Overall, the gathered data indicate that the *atm* mutant was highly sensitive to NSC120686, both phenotypically and concerning the *TDP1α* gene expression profiles. Alternatively, the upregulation of *TDP1β* in *e2fb*, *pol2a*, and *atr* supports its implication in the replication stress response. **Conclusions:** The current study demonstrates that genotoxic stress induced by CPT and NSC has a genotype-dependent effect reflected by a differential expression of *TDP1* genes and early phenotypic development.

## 1. Introduction

The high use of chemicals derived from anthropogenic activities, even though useful up to a certain point, can cause harm to the environment especially when improperly handled or discarded. When dispersed, these chemicals can come in contact with living organisms and contribute to inducing various types of stresses that can affect their genetic materials [1,2,3,4,5]. Modifications in the genetic material occur either as errors introduced during DNA replication, recombination, and even repair, or as base alterations caused by a wide range of chemical and physical agents. The latter type is defined as genotoxic stress, induced by genotoxins or mutagens, that results in DNA damage caused by physical (e.g., ultraviolet and ionizing radiation) or chemical (e.g., methyl methanesulfonate, bleomycin, and heavy metals) agents [6,7,8,9]. Given their sessile way of life, plants are constantly exposed to these genotoxic stresses. If the DNA damage is not properly repaired, genome instability occurs, with negative consequences on plant development and productivity [10,11,12,13]. The DNA damage response (DDR) pathway has evolved to maintain genome stability and ensure an appropriate DNA repair and cell cycle progression. DDR is an intricated signal transduction network—containing sensors, transducers, mediators, and effectors—coordinately acting to regulate downstream pathways that, in plants, include DNA repair, cell cycle checkpoints, programmed cell death (PCD) and endoreduplication [14,15,16,17]. After damage recognition by members of the MRN (MRE11, Rad50, and NBS1) complex, in the case of double-strand breaks (DSBs), or RPA (Replication Protein A) and POL2ε (DNA polymerase ε) for single-stranded DNA (ssDNA), DDR activation relies on the ATM (Ataxia Telangiectasia Mutated) and ATR (ATM and Rad3-related) protein kinase transducers [15,16]. *A. thaliana* ATM and ATR homologs have been isolated since the early 2000s [18,19]. The generation of *atm* and *atr* mutants demonstrated that both are hypersensitive to DSBs induced by γ-irradiation whereas ATR is also required for replicative stress [20]. ATR and ATM converge to activate the SOG1 (Suppressor of gamma response 1) transcription factor, acting as a master regulator of plant DDR [21,22]. SOG1 was initially discovered using a screening strategy to select suppressor mutants of the ionizing radiation (IR)-induced cell-cycle arrest [23]. Arabidopsis *sog1* mutants exposed to IR are defective in the induction of many genes [22], often leading to a phenotype similar to the *atm* mutant [14,19].

Systems alternative to the action of SOG1 are also present in plants. Evidence of the existence of this alternative cascade came from the genetic analysis of *wee1/sog1* double mutants which showed enhanced sensitivity to replicative stress compared to the *sog1* mutant [24]. Single and double mutants exposed to different types of genotoxic stresses, including hydroxyurea (HU) or mitomycin C (MMC), demonstrated that SOG1 and WEE1 can independently control the replication stress checkpoint [25]. The hypothesis of a SOG1-independent pathway is also supported by the analysis of *A. thaliana* mutants with partial deficiency in the replicative DNA pol ε, highly sensitive to replicative stress [20]. Different *pol2a* mutants (formerly known as *abo4-1*) have been isolated and also characterized as being hypersensitive to abscisic acid (ABA) and aphidicolin, an inhibitor of replicative polymerase [20]. Additional SOG1-independent pathways can involve the E2F-RBR1 (E2 transcription factor–RetinoBlastoma Related 1) complex, controlling the G1-to-S transition checkpoint [26]. The use of double- and triple-mutant combinations between SOG1 and E2F transcription factors demonstrated that E2FB is mostly involved in regulating plant growth under replication stress, by working antagonistically or synergistically with SOG1 [26].

Within the DDR downstream pathways, TDP1 (tyrosyl-DNA phosphodiesterase 1) functions as a DNA repair protein, being mainly involved in base excision repair (BER) and DNA-protein crosslink repair (DPCR) [27,28,29]. Its function is to hydrolyze the phosphodiester bond formed between the tyrosine residue of DNA topoisomerase I (TopI) and the 3′-phosphate of DNA [30,31]. Two *TDP1* genes are present in plants, namely, *TDP1α* and *TDP1β* [32], with *TDP1α* being the most studied and reported to have an active role in maintaining genome stability [27,33,34]. Phylogenetically, while TDP1α can be considered of non-plant origin as it is also present in other kingdoms [35,36], TDP1β is plant-specific since it was found to have originated from ancient vascular plants, still represented nowadays by species like *Amborella trichopoda* and *Selaginella moellendorffii*, where both sequences were present for the first time [37]. Plants defective in the *TDP1α* gene function are characterized by impaired growth and sensitivity to genotoxic stresses [27,34,38,39]. TDP1β is characterized by the presence of a HIRAN (HIP116, Rad5p, N-terminal) domain between the HKD (histidine, lysine, and aspartate) catalytic sites, while at the level of gene expression, it was shown to be ubiquitously expressed in all plant tissues and developmental stages as well as in the response to different types of stresses [40,41]. Recent data-mining studies indicate that the two genes may have both common and divergent functions [37,42,43]. Considering the knowledge deficit still surrounding the plant *TDP1* genes, the aim of this study was to investigate how *TDP1α* and *TDP1ß* are related to DDR. In order to do so, we used an experimental system composed of multiple single and double DDR mutants (*sog1*, *e2fb*, *pol2a*, *atm*, *atr*, *sog1/e2fb*, *sog1/pol2a*, and *pol2a/e2fb*) treated with genotoxic agents specifically affecting the TDP1 function, such as the NSC120686 substrate mimetic [44], and camptothecin (CPT), an inhibitor of TopI [31].

## 2. Materials and Methods

### 2.1. Plant Material and Treatments

Seeds used in this work included multiple *A. thaliana* DDR mutants along with the wild-type (WT) ecotype Col0. The DDR mutant collection comprises *sog1*, *pol2a*, *e2fb*, *atm*, and *atr*, along with the double mutants *sog1/pol2a*, *sog1/e2fb*, and *pol2a/e2fb*. The *sog1* mutant (known as *sog1-1*) was originally isolated in the Ler background and introgressed in the Col-0 background by Yoshiyama et al. [21]. The isolation of the *pol2a* (formerly known as *abo4-1*) mutant was reported by Yin et al. [45] and it was further characterized by Pedroza-Garcia et al. [20] whereas the *e2fb* mutant was obtained and characterized by Nisa et al. [26]. The *atr* (SALK_083543C) and *atm* (SALK_040423C) T-DNA insertion mutant lines were described in Garcia et al. [18] and Culligan et al. [19], respectively. The double mutants *sog1/pol2a*, *sog1/e2fb*, and *pol2a/e2fb* were obtained from the Institute of Plant Sciences Paris-Saclay (IPS2), Université Paris-Saclay, where they were produced by crossing the respective single mutants.

To grow the plants in vitro, seeds were surface-sterilized as follows: seeds were soaked in a tube containing 20% commercial bleach and subsequently soaked with 70% EtOH. Seeds were then washed with sterile water and sown in Petri dishes. The medium used for the in vitro cultivation was based on ½ MS (Murashige & Skoog, Duchefa Biochemie, Haarlem, The Netherlands) with 0.6% plant agar (Duchefa Biochemie).

The treatments used in this study included 50 nm campthotecin (CPT, Sigma-Aldrich, Milan, Italy) and 25 μM NSC120686 (provided by National Cancer Institute, Bethesda, MD, USA) which were added to the ½ MS medium. The concentrations were selected based on previous experiments [43]. Since these agents were dissolved in 0.5% DMSO (Sigma-Aldrich, Milan, Itlay), additional MS plates containing 0.5% DMSO were also used as control.

Petri plates containing the ½ MS medium and respective treatments were used for sowing the Arabidopsis seeds. For each treatment, three replicates of 100 seeds each were used. The plates were incubated in a cold chamber at 4 °C for 2 days and then moved to a temperature-controlled growth chamber at 25 °C, with a photon flux density of 150 μmol m^−2^ s^−1^, a photoperiod of 16/8 h light/dark, and 70–80% relative humidity inside the containers. The Petri plates were maintained under these conditions for 20 days.

### 2.2. Plant Phenotyping Analyses

To evaluate germination, seeds with a protrusion of the primary radicle were considered germinated, and the final germination percentage (G%), as well as mean germination time (MGT) were calculated [46]. Values are expressed as mean ± SD of three independent replicates with 100 seeds for each replica.

Seedling morphology was examined by measuring the radicle and aerial part length, along with the number of leaves in 20-day-old seedlings. Plantlets were photographed and each image was analyzed with the ImageJ software (https://imagej.nih.gov/ij/index.html). For radicle length, only the primary root was considered while for the aerial part, the length was measured from the root junction to the leaf apex. For these analyses, 20 plantlets were evaluated for each replicate.

### 2.3. RNA Extraction and cDNA Synthesis

RNA from treated/untreated 20-day-old seedlings was isolated using TRIZOL (Fisher Molecular Biology, Milan, Italy), according to the manufacturer’s suggestions. Approximately 100 seedlings were groundin liquid nitrogen, mixed with 750 μL Trizol and 150 μL Chloroform, and centrifuged for 15 min at 12,000× *g* and 4 °C. Isopropanol was added to the supernatant, followed by a centrifuge step for 10 min and washes with ethanol, 70% and 100%. In the end, 25 μL DEPC (diethylpyrocarbonate) water was added to resuspend the RNA pellet, which was subsequently treated with DNAse (Thermo Fisher Scientific, Milan, Italy) and quantified using a NanoDrop spectrophotometer (Biowave DNA, WPA, ThermoFisher Scientific, Waltham, MA, USA).

The complementary DNA (cDNA) was obtained using the RevertAid First Strand cDNA Synthesis Kit (Thermo Fisher Scientific), used as per the protocol suggested by the manufacturing company. A mix composed of 1 μL RevertAid Reverse Transcriptase (RT), 1 μL RiboLock RNase inhibitor (which protects the RNA templates from degradation), 4 μL 5X Reaction Buffer, 2 μL dNTPs mix, and 1 μL Random Primer was obtained and mixed with the DNase-treated RNA samples. The 20 μL solution was incubated at 37 °C for 30 min, followed by the addition of 1 µL 50 mM EDTA and subsequent incubation at 65 °C for 10 min.

### 2.4. Quantitative Real-Time PCR (qRT-PCR)

The qRT-PCR reactions were carried out with Maxima SYBR Green qPCR Master Mix (2X) (Thermo Fisher Scientific) using a Rotor-Gene 6000 PCR apparatus (Corbett Robotics Pty Ltd., Brisbane, QC, Australia) with the following amplification conditions: denaturation at 95 °C for 10 min, 45 cycles of 95 °C for 15 s, 60 °C for 30 s, and 72 °C for 30 s. Primers for the reaction were designed using the Primer3Plus program (http://primer3plus.com) and then further validated using the software IDT Oligo Analyzer (http://eu.idtdna.com/calc/analyzer).

The investigated genes were *TDP1α* (Accession no. AT5G15170) and *TDP1β* (Accession no. AT5G07400). Relative quantification was carried out using ubiquitin 4 (*UBQ*, Accession no. AT5G20620) as the reference gene [47]. The sequence of oligonucleotides used was the subsequent: FW-TDP1α—CGGTGACGGAGAGAGAAAGA, REV-TDP1α—GGACAAAAACGACGAATGGC, FW-TDP1β—TCACCTTGTTGCTTCAGTGC, REV-TDP1β—CCAGTCGTTTCAGTTGTGCT, FW-UBQ—TCATTTGGTGCTTCGTCT, REV-UBQ—GTCTCTGCTGATCTGGTG. The raw, background-subtracted fluorescence data provided by the Rotor-Gene 6000 Series Software 1.7 (Corbett Robotics) were used to estimate PCR efficiency (E) and threshold cycle number (Ct) for each transcript quantification. The Pfaffl method [48] was used for the relative quantification of transcript accumulation. For each sample, three replicates were used, and data are represented as a heatmap using the mean values of the triplicates. To distinguish between the effect of the DMSO solvent and CPT and NSC treatments, in these cases, data are represented as fold change (FC) to DMSO.

### 2.5. Statistical Analyses

Statistical analyses were conducted using the two-way analysis of variance (ANOVA), where the significance of mean differences was determined by Student’s *t*-test at a threshold of *p*-value ≤ 0.05.

## 3. Results

### 3.1. Germination Is Affected in the Mutants but Not in Response to Stress

**Figure 1:**
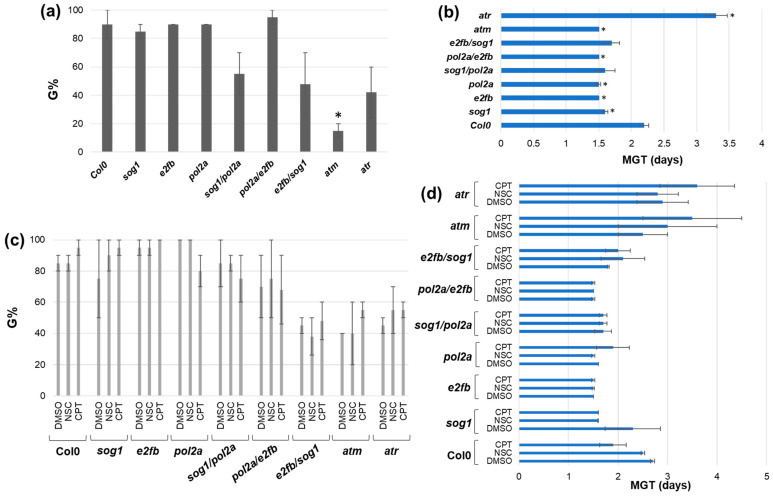
Germination parameters evaluated in *A. thaliana* Col0 and DDR mutants in the presence/absence of genotoxic stress. (**a**) Germination percentage (G%) in the absence of stress. (**b**) Mean germination time (MGT) in the absence of stress. Statistically significant differences (*p* < 0.05) from the Col0 control are indicated with an asterisk (*). (**c**) Germination percentage (G%) in the presence of CPT and NSC treatments, compared to the DMSO control. (**d**) Mean germination time (MGT) in the presence of CPT and NSC treatments, compared to the DMSO control. DMSO, dimethyl sulfoxide 0.5%; NSC, 0.025 mM NSC120686; CPT, 25 nm camptothecin.

As part of the phenotyping analyses of DDR mutants and genotoxic stress response, a first step was represented by the evaluation of germination perfomance (Figure 1).

In the absence of treatments, a significant decrease in the germination percentage (G%) was observed only in the *atm* mutant (Figure 1a). Regarding germination speed, the MGT parameter indicated that only the *atr* mutant germinated slower that Col0 while the other mutants germinated faster (Figure 1b).

When the seeds were germinated in the presence of CPT and NSC genotoxic agents, no statistically significant differences were observed compared to the DMSO controls for any of the mutants either in terms of percentage (Figure 1c) or speed (Figure 1d).

It is important to underline that since the genotoxic agents used in this study require to be suspended in DMSO, the control used for the statistical analysis regarding the effect of the treatments had to be referred to this compound as a control. The concentration of DMSO used was based on previous studies in *A. thaliana* [43,49,50,51]. However, slight differences between physiological conditions (MS1/2 medium only) and DMSO controls can be observed in some cases. In particular, the *pol2a* and *atm* mutants appeared to have a better germination (reaching 100%) when DMSO was added to the growth medium. Some studies reported that, in mammalian cells, the addition of DMSO to the growth medium reduces the formation of DSBs following IR-induced stress, due to its ability to act as an effective radical scavenger [52,53,54].

### 3.2. Changes in the Plant Growth Patterns Are Caused by Genotoxic Treatments

**Figure 2:**
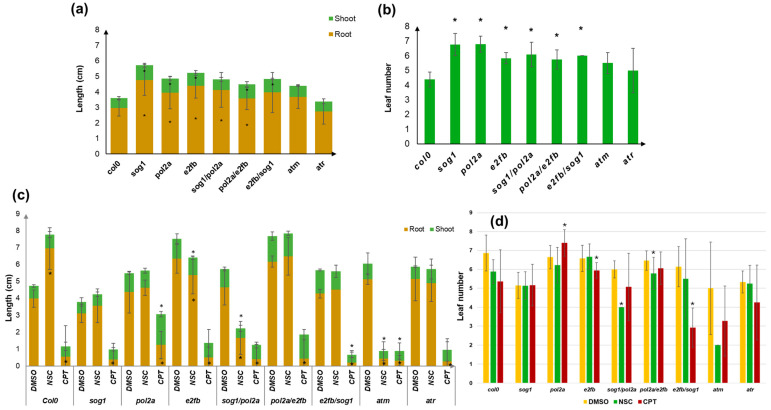
Phenotypic evaluation of DDR mutants and control Col0 lines in the presence/absence of genotoxic treatments. (**a**) Shoots (green) and roots (brown) length in the absence of genotoxic stress. (**b**) Leaf number in the absence of stress. Statistically significant differences (*p* < 0.05) from the Col0 control are indicated with an asterisk (*). (**c**) Shoot and root length in the presence of CPT and NSC treatments, compared to the DMSO control. (**d**) Leaf number in the presence of CPT and NSC treatments, compared to the DMSO control. Statistically significant differences (*p* < 0.05) from the DMSO control are indicated with an asterisk (*). DMSO, dimethyl sulfoxide 0.5%; NSC, 0.025 mM NSC120686; CPT, 25 nm camptothecin.

Despite the fact that the final germination percentage was not generally affected by the imposed treatments, the response of the DDR mutants in terms of seedling growth and development presented multiple changes (Figure 2). In the absence of stress, the majority of the investigated mutants (except for *atm* and *atr*) showed an improved growth compared to Col0, with longer roots, better developed shoots (Figure 2a), and more leaves (Figure 2b). This may be due to the fact that, for these mutants, we also observed faster germination, thus bringing the plant to a more advanced state of development compared to the Col0 line.

Following exposure to genotoxic stress, the Col0 line was negatively affected by the CPT treatment while root growth was improved by the NSC treatment (Figure 2c). The CPT treatment strongly inhibited root growth in all tested mutants, while in some cases (*e2fb/sog1* and *atm*), shoot growth was also significantly affected. Interestingly, despite the short radicle, in the *pol2a* mutant, shoot length was higher than that of the DMSO control. Also here, it should be noted that, in some cases, the use of DMSO alone had slight positive or negative effects compared to the standard conditions, thus strengthening the necessity to use this treatment as a control. This is in line with other studies showing either a priming or a cytotoxic effect in other plants, mostly related to the dosage or species [55,56,57]. Among the DDR mutants, the NSC treatment resulted in decreased root and shoot growth only for *e2fb*, *sog1/pol2a*, and *atm*, with the latter being the most affected by this agent. Regarding leaf number, the CPT treatment resulted in a significant reduction compared to the DMSO control for the *pol2a* and *e2fb/sog1* mutants while for the *e2fb* mutant slightly more leaves were counted (Figure 2d). The NSC treatments mostly affected the *sog1/pol2a* double mutant and only slightly the *pol2a/e2fb* double mutant.

**Figure 3:**
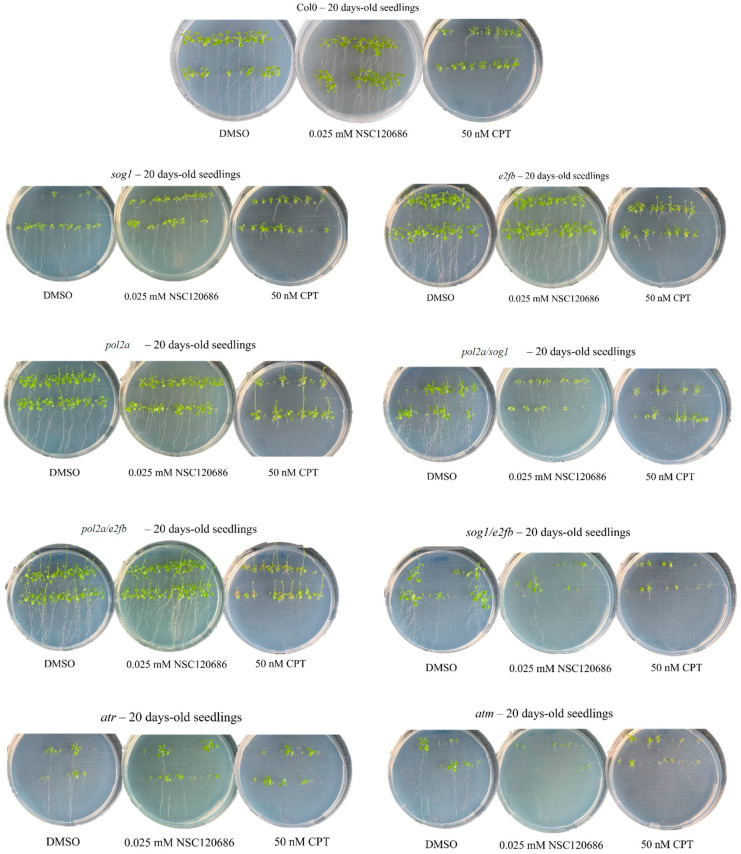
Representative images of *A. thaliana* 20-day-old plants belonging to DDR mutants *sog1*, *abo4-1*, *e2fb*, *abo4-1/sog1*, *abo4-1/e2fb*, *sog1/e2fb*, *atm*, and *atr* and the Col0 line grown in plates with MS1/2 supplemented with DMSO (0.5%), CPT (50 nM), or NSC120686 (0.025 mM).

To better define the effects of the treatments on the investigated materials, the overall phenotypes observed in the 20-day-old seedlings from Col0 and the DDR mutant lines are summarized in Table 1. The formation of branched roots (B) was observed under DMSO in almost all the lines (except for *sog1*), as well as in some cases after the NSC treatment, except for the *sog1*, *pol2a/sog1*, and *atm* mutants. Generally, CPT caused a decrease in the radicle growth in all the lines, giving rise to a short radicle phenotype (R). The *pol2a*, *pol2a/e2fb*, and *pol2a/sog1* mutants displayed an abnormal phenotype marked by the increased elongation of the flowering stem (F) in both the DMSO- and CPT-treated seedlings, which may raise the question whether the absence of *pol2a* could be responsible for this phenotype. The *pol2a/e2fb* mutants showed another peculiar phenotype represented by the presence of brown leaves (O). Examples of plantlets representative of each mutant and applied treatment are presented in Figure 3.

### 3.3. The Expression of TDP1α and TDP1βGenes Is Influenced by Both Genotype and Treatments

**Figure 4:**
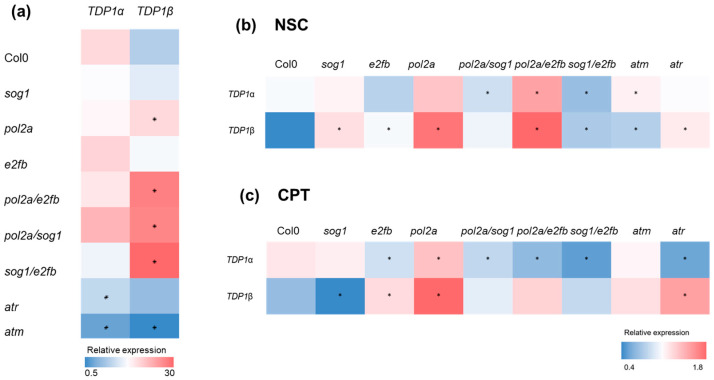
Heatmap representation of *TDP1α* and *TDP1β* relative expression values obtained from 20-day-old *A. thaliana* DDR mutants and the Col0 line. (**a**) Gene expression under control conditions in the absence of stress. (**b**) Gene expression in response to 0.025 mM NSC120686 (NSC) treatments. (**c**) Gene expression in response to 25 nm camptothecin (CPT). Values for NSC and CPT are presented as fold change (FC) to DMSO control. Statistically significant differences (*p* < 0.05) to the Col0 control are indicated with asterisks (*).

The *TDP1α* and *TDP1β* gene expression profiles were evaluated in Col0 and the *A. thaliana* mutant lines *sog1*, *pol2a*, *e2fb*, *pol2a/sog1*, *pol2a/e2fb*, *sog1/e2fb*, *atm*, and *atr*. A qRT-PCR was performed using seedlings grown in the absence/presence of stress induced by the genotoxic agents CPT and NSC (Figure 4). The gathered data show that in the seedlings grown in the absence of stress (Figure 4a), the *TDP1α* transcript was more expressed than *TDP1β* in the Col0 line. Moreover, both genes were significantly downregulated in *atm*, while *TDP1α* was also downregulated in the *atr* mutants compared to Col0. While the expression of *TDP1α* has not significantly changed in the other mutants, the *TDP1β* gene was differentially modulated in other mutants too. Namely, *TDP1β* was highly expressed in *pol2a* as well as in all double mutants (Figure 4a).

With respect to the data obtained from the NSC-treated seedlings (Figure 4b), it is possible to observe that *TDP1α* was significantly upregulated in the *pol2a/e2fb* and *atm* mutants, while for the *pol2a/sog1* and *sog1/e2fb* double mutants, the gene was downregulated. Differently, the *TDP1β* gene was significantly upregulated in all mutants, except for the *pol2a/sog1* background.

When considering the CPT treatment (Figure 4c), the *TDP1α* gene appeared to be upregulated in the *pol2a* mutants and downregulated in *e2fb* and *atr* as well as in all the double mutants. Also in this case, the *TDP1β* gene had a different behavior, being downregulated in the *sog1* mutant and upregulated in the *e2fb*, *pol2a*, and *atr* mutants.

Overall, looking at the expression profile of the two genes, it can be stated that these are differentially expressed in the different DDR mutants as well as in response to the imposed treatments. While *TDP1α* is highly influenced by CPT, the *TDP1β* gene is more impacted by the NSC treatment. Similarly, among the different mutants, the *TDP1α* expression is more conditioned by the *atm* and *atr* backgrounds while *TDP1β* appears to be linked to the *POL2A* functions.

## 4. Discussion

Extensive data regarding the implication of DDR genes in genotoxic stress response are available mainly when using DSB-inducing agents like γ-irradiation and zeocin [11,22,58,59], or compounds that induce replication stress [24,26,60]. The novelty brought by the current study consists in using agents that induce different types of DNA damage, as in the case of CPT, known to cause the formation of DNA-protein crosslinks [31,34], and the NSC120686 compound, acting as a TDP1 substate mimetic [44]. Whereas CPT is a TopI inhibitor that intercalates between DNA breaks, forming covalent complexes subsequently repaired by TDP1 [31], NSC is known as a substrate mimetic used to suppress the TDP1 activity [44,61]. Here, these compounds were used to treat an array of *A. thaliana* single and double mutants defective in DDR functions, including DNA polymerase ε (POL2A), which triggers ATR-dependent signaling leading to SOG1 activation [20], ATM and ATR transducers, the SOG1 master regulator [14,15,16], and the SOG1-independent pathway including the E2FB transcription factor [26]. This study included an initial phenotyping of these mutants in the presence/absence of CTP or NSC, followed by a gene expression study where *TDP1α* and *TDP1β* were investigated to understand how these genes are influenced by the DDR context.

Even if the germination performance of the investigated mutants was not affected by the genotoxic treatments (except for the germination speed in some cases), the subsequent plant development was altered in a treatment- and genotype-specific manner. A highly significant decrease in plant growth was observed mainly for the CPT treatment, while this was less evident for NSC (except for the *atm* and *sog1/pol2a* mutants). Considering the genotype-dependent effect, a finding worth noting relates to the *pol2a* single and double mutants which presented a phenotype marked by the increased elongation of the flowering stem together with a short root. A recent study identified POL2A as an ATM suppressor under topological stress applied using CPT [62]. The study reported that specific mutations in *A. thaliana* POL2A protein sequence could rescue the abnormal root growth of the *atm* mutants under CPT treatments, while the *pol2a* mutants themselves showed resistance to this treatment. The role of POL2A in promoting the S-phase progression and accelerating replication has been proposed [62], which may also be related with the elongation of the flowering stem observed in our study. Regarding the use of the NSC compound for plant research, all data available so far come from our previous works, evidencing that it is genotoxic, causing the accumulation of DNA damage both in vitro and in vivo conditions [41,43,57]. However, from a phenotypic point of view, this type of damage is either less pronounced compared to CPT, or it is more rapidly repaired [43,57]. When *A. thaliana tdp1* mutants were treated with NSC, a decrease in plant growth was observed and this was associated with the accumulation of SSBs and other associated lesions, rather than DSBs [43]. The fact that the *atm* and *sog1/pol2a* mutants were mainly affected by this treatment is puzzling and will require further evaluation.

The use of CPT and NSC treatments was driven by the fact that these compounds can have a direct or indirect effect on the function of the *TDP1* genes. Mechanistically, based on the existing literature, CPT administration results in blocking the topoisomerase I enzyme and the formation of TopI-DNA covalent complexes [63,64,65], which lead to DNA damage accumulation and the inhibition of plant growth [66,67]. In this situation, *TDP1* genes were reported to be downregulated in other plant models [57]. Differently, when providing NSC, the TDP1 enzyme is prevented from engaging with its substrate due to the presence of the mimicking compound [44], thus still leading to the accumulation of TopI-DNA covalent complexes and DNA damage but to a lesser extent. In relation to this case, the *TDP1* genes became more active, in an attempt to enhance protein production and compensate for its binding to the mimicking substrate [57]. Nonetheless, the observed genotype-dependent difference in the expression of *TDP1α* and *TDP1β* indicate that their function could be conditioned by the respective DDR genes. The obtained data revealed that the *TDP1β* gene was more expressed in the *pol2a* mutated background already in the absence of stress. This is indicative of the role of the *TDP1β* gene in replication stress, in agreement with recent findings [43]. Differently, the downregulation of *TDP1α* expression in both *atm* and *atr* suggests the role of this gene in the repair of both DSBs and ssDNAs. This is also in line with evidence indicating that *TDP1α* depletion generally affects whole genome stability [27,28,33,34,38]. Regarding the effect of NSC on gene expression, *TDP1β* was upregulated in almost all mutants, while *TDP1α* was upregulated only in the *pol2a/e2fb* and *atm* mutants. This may indicate that the *TDP1β* gene may be ubiquitously required in the DDR context to repair the damage induced by NSC. Differently, in response to CPT, *TDP1α* was upregulated in all the *pol2a* mutants whereas *TDP1β* was upregulated in the *e2fb*, *pol2a*, and *atr* mutants. Thereby, when DNA-protein crosslinks are formed in these mutants, replication-coupled repair mechanisms (combining POL2A, ATR, and E2FB) are at work to remove the induced errors. The activation of this mechanisms in response to CPT was reported also in other types of mutants [26,43], upstream of the DNA repair pathways required to remove diverse different types of DNA adducts [28]. To further reason on these differences, a recent study using *A. thaliana tdp1α* and *tdp1β* mutants challenged with genotoxic stresses demonstrated that CPT and NSC mostly affected the *tdp1α* lines while *tdp1β* was more susceptible to other agents like cisplatin, curcumin, and zeocin, strengthening the connection of the *TDP1β* gene with the replication machinery and DSB repair [43].

Considering that these types of DNA damage can also result from environmental stresses (mainly abiotic, like exposure to heavy metals, UV, and high temperature), understanding the roles that these genes play in DDR can also lead to practical applications in agriculture. For instance, the high expression of the *TDP1α* gene during eggplant seed imbibition has been associated with enhanced germination performances, thereby potentially serving as a molecular indicator of seed quality [68]. More recently, a genome-wide association study performed in peanuts reported that *TDP1*(α) was associated with a QTL involved in drought tolerance [69]. Moreover, key DDR components play pivotal roles in any stress response. Incorporating DDR knowledge into breeding strategies, combined with biotechnological tools like CRISPR/Cas9, could ultimately lead to the enhancement of crop resilience [70,71,72]. This integrated approach holds promise for sustainable food production in the face of climate change, optimizing yields and ensuring the stability of global food supplies.

## 5. Conclusions

In conclusion, the current study demonstrated that genotoxic stress induced by camptothecin and NSC120686 has a genotype-dependent effect when considering a panel of *A. thaliana* mutants defective in DDR genes. When the expression of *TDP1α* and *TDP1β* genes was evaluated in this context, the hereby described results indicate that while *TDP1α* may be more involved in the repair of DSBs and ssDNAs, *TDP1β* functions could be mostly related to replication stress. Nonetheless, further studies are needed to elucidate the complex relations between *TDP1* and DDR players. These could be designed to include treatments with other genotoxic agents and availing of a more complete mutant collection. A better comprehension of the roles and functions of *TDP1α* and *TDP1β* genes in the context of plant DDR can lead to an improved understanding of the mechanisms that make plants able to deal with the constant exposure to damaging agents while fighting to maintain their genome integrity.

## Figures and Tables

**Table 1:** Classification of observed phenotypes in DDR mutants and Col0 lines under genotoxic stress. B, branched roots; R, short roots; F, floral stem; O, brown leaves.

	DMSO	NSC	CPT
*col0*	B	B	R
*sog1*			R
*pol2a*	F, B	B	F, R
*e2fb*	B	B	R
*sog1/pol2a*	F, B		F, R
*pol2a/e2fb*	F, B	F, B	F, R, O
*e2fb/sog1*	B	B	R
*atm*	B	R	R
*atr*	B	B	R

## Data Availability

The original contributions presented in the study are included in the article; further inquiries can be directed to the corresponding author.
